# COVID-19 in Russia: Clinical and Immunological Features of the First-Wave Patients

**DOI:** 10.32607/actanaturae.11374

**Published:** 2021

**Authors:** T. V. Bobik, N. N. Kostin, G. A. Skryabin, P. N. Tsabai, M. A. Simonova, V. D. Knorre, O. N. Stratienko, N. L. Aleshenko, I. I. Vorobiev, E. N. Khurs, Yu. A. Mokrushina, I. V. Smirnov, A. I. Alekhin, A. E. Nikitin, A. G. Gabibov

**Affiliations:** Shemyakin-Ovchinnikov Institute of Bioorganic Chemistry, Russian Academy of Sciences, Moscow, 117997 Russia; Central Clinical Hospital of the Russian Academy of Sciences, Moscow, 117593 Russia; Engelhardt Institute of Molecular Biology, RAS, Moscow, 119991 Russia

**Keywords:** serological analysis of patients with COVID-19, SARS-CoV-2-specific antibody subclasses

## Abstract

The coronavirus disease outbreak in 2019 (COVID-19) has now achieved the level of a global pandemic and affected more than 100 million people on all five continents and caused over 2 million deaths. Russia is, needless to say, among the countries affected by SARS-CoV-2, and its health authorities have mobilized significant efforts and resources to fight the disease. The paper presents the result of a functional analysis of 155 patients in the Moscow Region who were examined at the Central Clinical Hospital of the Russian Academy of Sciences during the first wave of the pandemic (February–July, 2020). The inclusion criteria were a positive PCR test and typical, computed tomographic findings of viral pneumonia in the form of ground-glass opacities. A clinical correlation analysis was performed in four groups of patients: (1) those who were not on mechanical ventilation, (2) those who were on mechanical ventilation, and (3) those who subsequently recovered or (4) died. The correlation analysis also considered confounding comorbidities (diabetes, metabolic syndrome, hypertension, etc.). The immunological status of the patients was examined (levels of immunoglobulins of the M, A, G classes and their subclasses, as well as the total immunoglobulin level) using an original SARS-CoV-2 antibody ELISA kit. The ELISA kit was developed using linear S-protein RBD-SD1 and NTD fragments, as well as the N-protein, as antigens. These antigens were produced in the prokaryotic *E. coli *system. Recombinant RBD produced in the eukaryotic CHO system (RBD CHO) was used as an antigen representing conformational RBD epitopes. The immunoglobulin A level was found to be the earliest serological criterion for the development of a SARS-CoV-2 infection and it yielded the best sensitivity and diagnostic significance of ELISA compared to that of class M immunoglobulin. We demonstrated that the seroconversion rate of “early” N-protein-specific IgM and IgA antibodies is comparable to that of antibodies specific to RBD conformational epitopes. At the same time, seroconversion of SARS-CoV-2 N-protein-specific class G immunoglobulins was significantly faster compared to that of other specific antibodies. Our findings suggest that the strong immunogenicity of the RBD fragment is for the most part associated with its conformational epitopes, while the linear RBD and NTD epitopes have the least immunogenicity. An analysis of the occurrence rate of SARS-CoV-2-specific immunoglobulins of different classes revealed that RBD- and N-specific antibodies should be evaluated in parallel to improve the sensitivity of ELISA. An analysis of the immunoglobulin subclass distribution in sera of seropositive patients revealed uniform induction of N-protein-specific IgG subclasses G1–G4 and IgA subclasses A1–A2 in groups of patients with varying severity of COVID-19. In the case of the S-protein, G1, G3, and A1 were the main subclasses of antibodies involved in the immune response.

## INTRODUCTION

The pandemic, officially declared by the WHO on March 11, 2020, after the rapid spread of the new coronavirus disease in 2019 (COVID-19), has proved a challenge for the global medical and scientific communities. By February 2021, more than 100 million people had been infected with the severe acute respiratory syndrome coronavirus 2 (SARS-CoV-2) across the world, and more than 2 million people had died. The infection spread rather quickly in the regions of Russia. According to the Ministry of Health of the Russian Federation, as of February 10, 2021, a total of more than 4 million people have been infected across the country; of these, more than 80,000 people have died.

The new coronavirus SARS-CoV-2, which belongs to the genus *Betacoronavirus*, is a cytopathic singlestranded RNA virus assigned to the II pathogenicity group. This virus infects cells carrying angiotensinconverting enzyme 2 (ACE2) receptors on their surface, mainly type II alveolar pneumocytes and, to a lesser extent, other epithelial cells [1]. Infection with the SARS-CoV-2 coronavirus leads to a wide range of manifestations, from asymptomatic to severe acute respiratory distress syndrome (ARDS) leading to death. According to our statistical analysis, about 80% of patients have a mild form of the disease not requiring hospitalization, with clinical signs of an acute respiratory tract infection with typical catarrhal symptoms, and they usually develop spontaneous recovery. The disease course usually resembles that of an acute respiratory viral infection (ARVI) caused by the influenza A and B viruses, rhinoviruses, adenoviruses, and seasonal coronaviruses; however, in some cases, the SARS-CoV-2 virus infection can lead to a very rapid acute inflammation with the development of severe bilateral pneumonia, hemorrhagic fever, and organ dysfunctions. A dramatic course of the disease is accompanied by severe pneumonia and affects 15% of patients; about 5% of patients develop ARDS and multiple organ failure. The mortality rate varies from country to country and, according to recent data, amounts to 1.04 to 8.5% of confirmed disease cases. Over the past year, many attempts have been made to establish a relationship between various factors (e.g., gender, age, race, comorbidities, various indicators and markers (including genetic ones), etc.) and the severity of the disease [2-8].

However, despite the large amount of data accumulated to date, most of the identified correlations remain inconsistent. In most publications, the genetic predisposition to the development of complications is associated with the structural features of ACE2, antigen presentation system, and the genes responsible for the innate immune system [9].

Humoral responses were used as the main markers of disease severity in other viral lung infections, including SARS-CoV and influenza virus infections [10, 11, 12, 13].

The SARS-CoV-2 genome encodes four structural proteins: spike (S-protein), nucleocapsid (N-protein), envelope (E), and membrane (M) [14]. The S and N proteins are the two main coronavirus antigens that induce production of immunoglobulins [15]. Anti- N-protein antibodies are often induced in relatively higher amounts than other proteins used as the main targets of serological assays [15, 16].

The receptor binding domain (RBD), which is situated in the spike protein S1 subunit, is the main target of neutralizing antibodies (NAbs) and is also used in the design of vaccines [17, 18, 19, 20].

According to reported data (WHO statistics), the mortality rate in Russia (1.89%) remains one of the lowest in the world. This fact still requires a detailed investigation. Of course, factors related to the healthcare organization in the Russian Federation may play a role in this phenomenon; however, we may suggest that the explanation for this phenomenon is related to demographic factors, as well as factors associated with risk groups and markers of inflammation severity. It was of interest to characterize in detail the humoral responses of adaptive immunity in cohorts of patients in the Russian population in response to coronavirus infection.

## EXPERIMENTAL

**Materials**

In this study, we used reagents from Sigma, Bio-Rad, Thermo Scientific (USA), Pharmacia (Sweden), Difco (England), Panreac (Spain), and Reakhim (Russia).

**Preparation of recombinant proteins and SARS-CoV-2 S- and N-protein fragments**

Artificially synthesized DNA fragments encoding S-protein RBD (330–538 aa), RBD-SD1 (330–590 aa), NTD (17–305 aa) fragments, and the N-protein sequence (1–420 aa) of the SARS-CoV-2 virus were cloned into pET22b plasmids using NdeI and XhoI restriction endonucleases. The correctness of the produced constructs was confirmed by sequencing.

*Escherichia coli *BL21 (DE3) cells transformed with the produced genetic constructs were cultured in a 2YT medium with ampicillin at 37°C and vigorous stirring until OD_600_ = 0.4, induced with 1 mM IPTG, and cultured at 30°C for 6 h. Isolation and purification of recombinant proteins from inclusion bodies was performed using metal chelate chromatography (HiTrap FF, GE Healthcare, USA) under denaturing conditions.

Expression and purification of the recombinant RBD (amino acid residues 320–537) produced in the eukaryotic system of CHO cells was performed according to the previously described method [21].

## ELISA

The purified recombinant RBD, RBD-SD1, NTD, and N-protein produced in the prokaryotic *E. coli *system were adsorbed to plate wells using buffer containing 50 mM sodium bicarbonate and 4 M urea, pH 10.6. For the detection of SARS-CoV-2-specific antibodies, a mixture of RBD-SD1, NTD, and N antigens at a ratio of 40/20/40 ng per well for each antigen (100 ng) was placed into Nun MaxiSorp flat-bottom 96-well plates (Nunc, USA) and incubated at 4°C without stirring for 16 h. After incubation, the solution was removed from the wells, the wells were washed with distilled water, and a blocking solution (phosphate buffered saline, 0.1% Tween 20, 3% BSA) was added. The plates were incubated at room temperature without stirring for 1 h. At the end of the incubation, the blocking solution was removed and the plates were dried to dryness at room temperature and stored at 6 Ѓ} 2°C.

In experiments with biological samples, sera were diluted at a ratio of 1 : 100 (for the analysis of total N-protein-specific immunoglobulin G) or 1 : 10 (in the other cases) in a washing solution (phosphate buffered saline, 0.1% Tween 20), placed into the wells, and incubated at 37°C and stirring (700 rpm, 30 min). The plates were washed 5 times with a washing solution, and antibodies to the appropriate classes and subclasses of human antibodies were added in a conjugate dilution solution (phosphate buffered saline, 0.1% Tween 20, 0.1% BSA) and incubated at 37°C and stirring (700 rpm, 30 min) [22]. The plates were washed 5 times with a washing solution, and anti-species antibodies conjugated with horseradish peroxidase in a conjugate dilution solution were added and incubated at 37°C and stirring (700 rpm, 30 min). After washing the plates with a washing solution (5 times), the TMB substrate was added and incubated in the dark for 15 min. The reaction was stopped with a 10% phosphoric acid solution, and the absorbance was measured at a wavelength of 450 nm on a plate reader.

For a correct interpretation of ELISA results, the threshold ODcrit was calculated based on OD values of a panel of sera from healthy donors (OD-ref), using the average optical density in these wells according to the following formula:

ODcrit = OD-ref + 3 × standard deviations.

The resulting ODcrit value was used to calculate the positivity index for each test sample using the following formula:

PIsamp = ODsamp/ODcrit.

At PIsamp ≥ 1, a blood serum sample was considered positive (the sample contains SARS-CoV-2 coronavirus- specific antibodies); at PIsamp < 0.9, a serum sample was considered negative.

The data were statistically processed using the GraphPad Prism 8 software.**  **

## RESULTS AND DISCUSSION

This study was based on clinical material collected at the Central Clinical Hospital of the Russian Academy of Sciences during April–May, 2020. A total of 155 patients diagnosed with COVID-19 were examined. The criteria for inclusion in the group of COVID-19-positive patients were a positive PCR test and pulmonary lesions identified in CT scans as ground-glass opacities. We searched for statistically significant differences in the disease course among groups of patients different in gender, age, and comorbidities. The number of days spent in the hospital was used as an indicator to indirectly assess the disease severity.

**Fig. 1:**
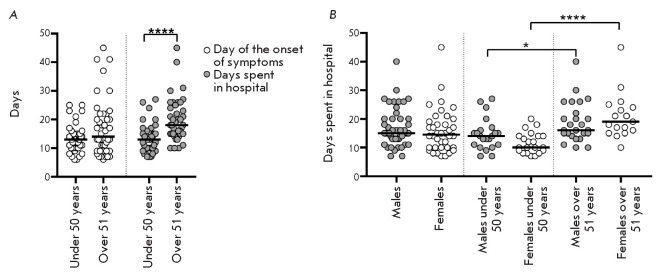
Distribution of the number of days from the onset of symptoms to hospitalization (A) and days spent in a hospital (*A*, *B*) among patients of different age groups

An analysis (*Fig. 1A*) using the nonparametric Mann–Whitney test revealed that the number of days after the onset of symptoms to hospitalization did not differ among patients of two age groups, and that there was no correlation between the number of days and the age of patients within the two age groups. However, the length of hospital stay was longer in the group of patients over 51 years of age (*p* < 0.0001), which indicates a more severe course of the disease in older patients. An analysis of the effect of gender on the length of hospital stay in patients of the two age groups (*Fig. 1B*) revealed slight differences between the groups of males under 50 and those over 51 years of age (*p *= 0.0177). There was a significant difference (*p* < 0.0001) in the length of hospital stay in females under 50 and those over 51 years of age. There were no statistically significant differences in the length of hospital stay in patients of different gender within the two age groups and regardless of age. The identified dependencies were confirmed by a correlation analysis; the Spearman’s correlation coefficient (*r*) value was statistically significant in the group of females (*r *= 0.65) and insignificant in the group of males (*r *= 0.31). An analysis of variance on the relationship between the length of hospital stay and a linear combination of age and gender factors showed that gender was not a significant factor (*p *= 0.719), while age, on the contrary, affected the length of hospital stay (*p * < 0.0001), and this relationship was more significant in the female group than in the male group (*p* < < 0.0001 and* p *= 0.0278, respectively). Therefore, this difference in the length of hospital stay of patients of different age groups is significantly associated with the difference between females of older (over 51 years) and younger (under 50 years) ages.

According to the published data [4, 5, 23, 26], comorbidities, such as hypertension, diabetes mellitus, and obesity, are risk factors for a severe course of COVID-19. We investigated the effect of these comorbidities on the length of hospital stay. We analyzed the length of hospital stay in three groups of patients: 1) with hypertension, diabetes mellitus, or obesity (HDO), 2) with other comorbidities, 3) without comorbidities.

**Fig. 2:**
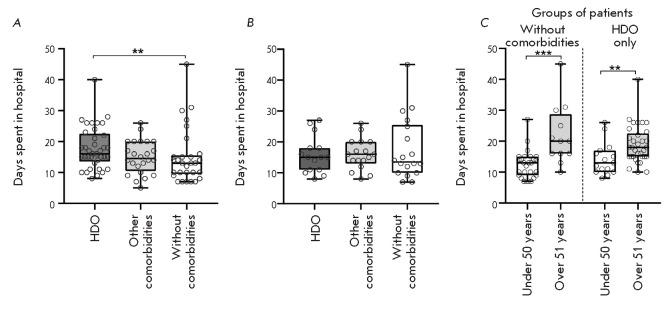
Distribution of the number of days spent in a hospital by patients with/without comorbidities (*A*–*C*) and patients of different age groups (*C*). (*A*) – groups of patients regardless of age; (*B*) – groups of patients with the same median age. (*C*) – comparison of the mean length of hospital stay in patients of age groups, with/without comorbidities. HDO – hypertension, diabetes mellitus, and obesity

The nonparametric Mann–Whitney test results revealed a significant excess (*p *= 0.01) in the length of hospital stay of patients in the group with comorbidities, such as hypertension, diabetes mellitus, and obesity, compared to that in the group without comorbidities (*Fig. 2A*). However, further analysis showed that the age median of patients in these groups was very different: 62 and 43 years, respectively. After matching the median between the groups by excluding patients of the maximum and minimum age, respectively, from the samples, there was no significant difference in the length of hospital stay between the groups (*Fig. 2B*). Comparison of the length of hospital stay in the HDO patients of the two age groups and in patients without comorbidities (*Fig. 2C*) revealed no effect of the diseases under consideration. The effect of age is statistically significant both in the group of patients without comorbidities (*p* = 0.0001) and in the group with these diseases (*p *= 0.0076). In this case, the length of hospital stay in patients of the same age groups, differing in the presence/absence of comorbidities, did not differ statistically significantly. Thus, there was no effect of comorbidities on the severity of COVID-19 in the studied cohort of patients. Perhaps, previously published data on a correlation between disease severity and some comorbidities did not consider the age imbalance in the compared groups.

**Fig. 3:**
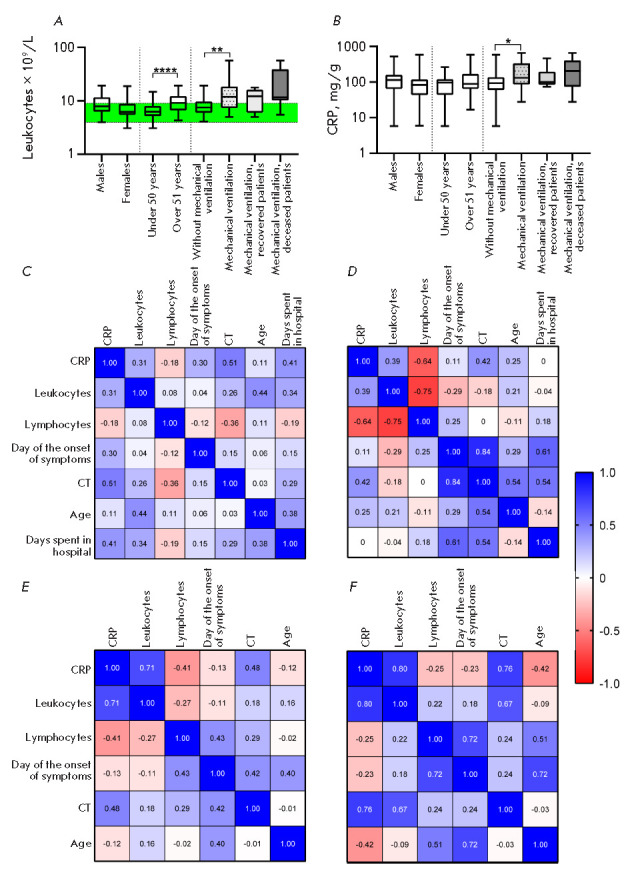
Distribution of the leukocyte count (*A*) and C-reactive protein level (*B*) within groups of patients of different ages, gender, and disease severity. The interval of normal values is marked in green. (*C*–*F*) – correlations for groups of patients who did not need mechanical ventilation (*C*), who needed mechanical ventilation (*n *= 16) (*E*), with subsequent recovery (n = 7) (*D*) or death (n = 9) (*F*)

To identify differences in some hematological characteristics among groups of patients with differing severity of COVID-19, the cohort of hospitalized patients was divided into those who needed and did not need mechanical ventilation. The results of clinical studies of patients requiring mechanical ventilation were analyzed either in total or in two groups, depending on the disease outcome (recovery or death). The nonparametric Mann–Whitney test revealed a significant (*p* < 0.0001) increase in the leukocyte count in patients of the older age group (over 51 years of age) compared to that in the group under 50 years of age (*Fig. 3A*). These data are consistent with the results of other studies [25, 26, 27].

increase in the leukocyte count was also detected in patients on mechanical ventilation (*p *= 0.006), which is consistent with previous data indicating that leukocytosis is associated with a severe course of COVID-19 and a high risk of death [25, 28, 29]. However, there was no significant differences in the leukocyte count between the groups of patients who recovered after mechanical ventilation therapy and those who died; therefore, it is erroneous to consider an increase in the leukocyte count as a prognostic factor of disease outcome. However, a number of researchers have proposed using leukocytosis in combination with idiopathic lymphopenia as a prognostic marker of disease severity. According to various sources, lymphopenia is detected in 40–80% of COVID-19 cases [30, 31, 32] and is pronounced in patients in critical condition [5, 33]. However, despite numerous studies indicating a close relationship between lymphopenia and disease severity, we did not find significant differences in the lymphocyte count in groups of patients with differing severity of COVID-19 in our cohort. Many researchers have suggested considering the C-reactive protein (CRP) as a prognostic factor [3, 36], a high level of which is associated with a worsening of the disease. A number of studies [3, 5, 37] have reliably demonstrated a significant increase in the blood CRP level in criticalcondition patients. However, some researchers have found a slight [38], or even no, difference [39] in the CRP level at different severities of the disease. Among the groups in our study cohort, a weak but statistically significant difference (*p *= 0.04) in the mean CRP concentration was found only between the groups of patients who underwent (or not) mechanical ventilation therapy (*Fig. 3B*).

A correlation analysis within four groups of patients: 1 – not on mechanical ventilation (*Fig. 3C*); 2 – on mechanical ventilation (*Fig. 3E*); 3 – those of them who subsequently recovered (*Fig. 3D*); or (4) died (*Fig. 3F*), revealed a correlation (from moderate to strong) in all groups between the CRP level and the degree of lung involvement assessed by CT. The CRP concentration in inflammatory diseases, including various pneumonias, was shown to correlate with the inflammation level and unaffected by factors such as age, gender, and the physical condition of the patient. CRP can be used to diagnose COVID-19 because the diagnostic sensitivity of CT alone is 76.4%, and CRP can detect inflammation in early pneumonia [40].

In groups of patients on mechanical ventilation, we found a significant correlation between the leukocyte count and CRP, moderate upon further recovery and strong upon death, which may indicate an intense inflammatory process.

The probability of two diametrically opposite outcomes of COVID-19 in severe patients on ventilation may be assessed through a correlation analysis. In recovered patients, there is further a strong correlation (*r *= 0.84) between the number of days after symptoms onset and the severity of lung involvement. Perhaps, due to impaired early antiviral immunity in these patients, a SARS-CoV-2 infection persists for a while and gradually increases the degree of damage to the lung tissue, until the patient is hospitalized due to symptoms associated with lung damage. In-hospital treatment, including mechanical ventilation, helps resolve the viral infection.

Currently, studies on humoral responses of adaptive immunity in a SARS-CoV-2 coronavirus infection are under way to determine whether there is a connection between the body’s immunological reactions and different scenarios of disease course, as well as the influence of various factors on them (gender, age, comorbidities, etc.). The inconsistency of data obtained over the past year necessitates further accumulation and a large-scale analysis. We compared qualitative and quantitative parameters of the B-cell immune response in different groups of patients diagnosed with COVID-19. Changes in the immune response were assessed by ELISA of blood serum samples from 155 patients with a confirmed diagnosis of COVID-19; of these, 105 patients were hospitalized at different times after the onset of symptoms. As antigens, we used a mixture of recombinant proteins, SARS-CoV-2 S-protein fragments (RBD-SD1 and NTD), and the recombinant N-protein, which were produced in the prokaryotic *E. coli *system and adsorbed in denatured state to plate wells.

**Fig. 4:**
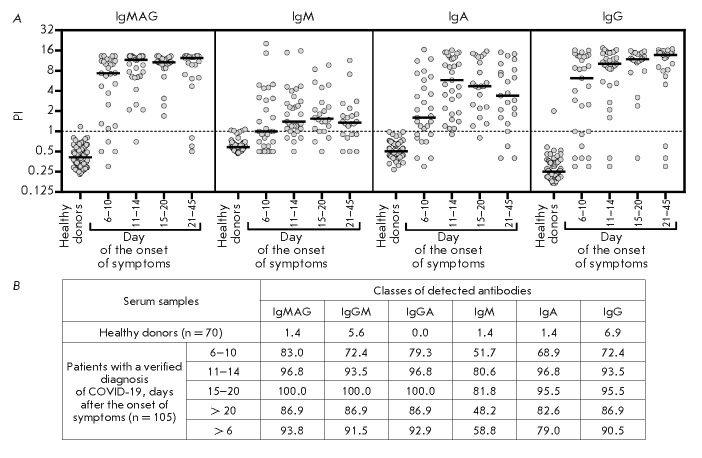
Results of serodiagnostic ELISA tests of blood sera from healthy donors and patients with a confirmed diagnosis of COVID-19 at different times after the onset of symptoms. (*A*) – individual values of the positivity index of test samples calculated upon detection of SARS-CoV-2-specific IgM, IgA, and IgG antibodies, separately or simultaneously. A mixture of recombinant proteins, SARS-CoV-2 S-protein RBD-SD1 and NTD fragments, and the recombinant N-protein was used as antigens. The sample positivity index was calculated as the sample signal to mean signal ratio for healthy donor samples (*n *= 70) + 3 standard deviations. The threshold value (PI = 1) is marked with a dashed line. (*B*) – number of samples exceeding the threshold value (expressed as %) for one or more of the indicated SARS-CoV-2-specific classes of antibodies

The assay results (*Fig. 4A*), expressed as the distribution of a calculated sample positivity index (PI) depending on the number of days after the onset of symptoms, revealed differences in the timing of the emergence of antibodies specific to the used SARS-CoV-2 fragments, depending on the time after the onset of symptoms. For class M, G, and A immunoglobulins, the median positivity index exceeding the threshold value (PI = 1) was reached on day 6 after the onset of symptoms. The maximum values were detected on day 11–14 for class A immunoglobulins, day 15–20 for class M immunoglobulins, and day 20 for class G immunoglobulins, which is consistent with the data obtained using other test systems [8, 41]. The maximum sensitivity of ELISA detection of IgG antibodies using our test system reached 95.5% in a range of 15–20 days after the onset of symptoms (*Fig. 4B*). In the case of the IgM and IgA antibodies, the maximum sensitivity of 81.8 and 96.7% was observed within 11–14 and 15–20 days after the onset of symptoms, respectively, and then it decreased, remaining significantly higher in the case of immunoglobulins A. A decrease in the sensitivity of detection of IgM and IgA antibodies by ELISA may be explained by a gradual decline in the levels of these antibodies in the bloodstream at a later follow-up period [42, 43]. The highest ELISA sensitivity (more than 93.8%) and specificity (98.6%) of detection of SARS-CoV-2-specific antibodies throughout the study period was achieved upon determination of total immunoglobulins M, G, and A. The sensitivity of detection of IgM, IgA, and IgG antibodies was slightly lower and amounted to more than 58.8, 79, and 90.5%, respectively. A ROC analysis was used to compare the diagnostic value of the tests at selected threshold levels. The AUC indicator was 0.93 (95% CI: 0.90–0.96) for a IgA analysis, 0.87 (95% CI: 0.83–0.92) for a IgM analysis, and 0.95 (95% CI: 0.93–0.98) for a IgG analysis. Since IgM and IgA antibodies have a similar timing of emergence and disappearance in the bloodstream, and the absolute values of sample positivity indices and the calculated sensitivity and diagnostic significance of ELISA are significantly higher for IgA antibodies than for IgM antibodies, it may be argued that detection of class A immunoglobulins is more reasonable for a diagnosis of COVID-19.

**Fig. 5:**
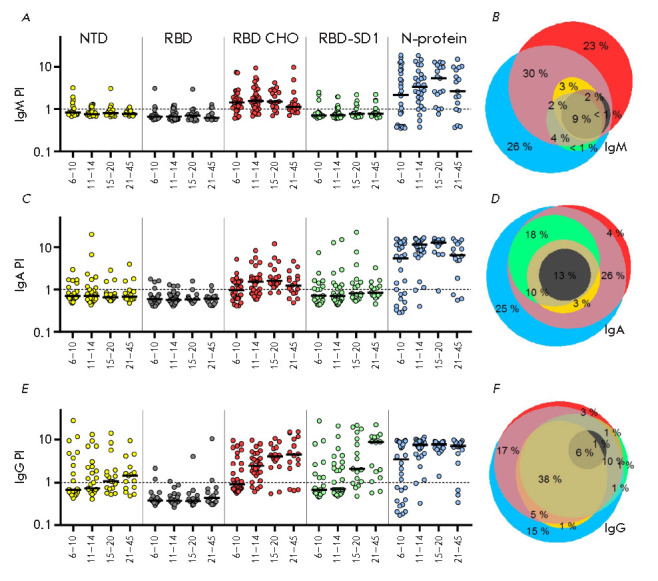
Results of serodiagnostic ELISA tests of blood serum samples from patients with a confirmed diagnosis of COVID-19 hospitalized at various times after the onset of symptoms. (*A, C, E*) – sample positivity index calculated upon detection of SARS-CoV-2 S-protein NTD, RBD, and RBD-SD1 fragment- and N-protein-specific IgM (*A*), IgA (*C*), and IgG (*E*) antibodies. (*B, D, F*) – Venn diagrams representing antigen-specificity spectra of IgM (*A*), IgA (*C*), and IgG (*E*) immunoglobulins in samples

To determine the contribution of each antigen to the ELISA sensitivity at different times after the onset of symptoms, we evaluated the level of antibodies specific to each of the antigens separately. As antigens in the analysis, we used S-protein RBD-SD1 and NTD fragments and the N-protein produced in the prokaryotic *E. coli *system and adsorbed in denatured state to plate wells. Similarly, the produced RBD fragment (RBD *E. coli*) was used to assess the contribution of SD1-specific immunoglobulins to the ELISA sensitivity. Recombinant RBD produced in the eukaryotic CHO system (RBD CHO) was used as an antigen representing the conformational RBD epitopes. The assay results (*Fig. 5A,C,E*) reveal a different timing of the emergence of antibodies, which depends on the antigen nature and the time after the onset of symptoms. The median positivity indices of N- and RBD (CHO)-specific class M and A immunoglobulins exceeded the threshold values on day 6 after the onset of symptoms, reached maximum values by day 11–14 in the case of RBD (CHO)-specific IgM antibodies and day 15–20 in other cases, and decreased after 3 weeks of observation. In the case of the antigens representing linear epitopes of the S-protein (RBD *E. coli*, RBD-SD1, and NTD), the number of seropositive patients in each time range did not exceed 10%, which did not allow the median positivity indices of immunoglobulins specific to these antigens to exceed the threshold. The seroconversion rate of SARS-CoV-2 N-protein-specific class G immunoglobulins is significantly higher than that of antibodies of other specificity; the median level of N-specific antibodies significantly exceeded the threshold value as early as on day 6 after the onset of symptoms, reaching a maximum on the second week. At the same time, the median level of RBD (CHO)-specific conformationdependent antibodies exceeded the threshold by the second week after the onset of symptoms, reaching its maximum within 21–45 days.

For IgG antibodies specific to NTD and RBD-SD1 antigens containing linear epitopes, the threshold value was exceeded only on the third week after the onset of symptoms. Thus, the seroconversion rate of early IgM and IgA antibodies is somewhat higher for antibodies specific mainly to the conformational RBD fragment epitopes than for N-specific antibodies. Conversely, the seroconversion rate of IgG antibodies decreased in the series of N-, conformation-dependent RBD (CHO)-, and conformation-independent RBD-SD1/NTDspecific antibodies. According to the obtained data (*Fig. 5*), the N-protein has the highest immunogenicity, as described earlier [44], while the linear RBD and NTD epitopes have the least immunogenicity. Thus, strong immunogenicity of the RBD fragment, reported previously [45], is mainly associated with conformational epitopes. Linear SD1 subdomain epitopes have strong but slowly developing immunogenicity, which may be especially important in light of the data on the existence of neutralizing antibodies specific to a linear epitope located in this region [45]. The spectra of antigen specificity were found to differ for class M, A, and G immunoglobulins (*Fig. 5B,D,E*). The number of seropositive patients with blood antibodies specific to only one “strong” immunogen was found to decrease in the series of class M, A, and G immunoglobulins and accounted for 49%, 29%, and 19% of the total seropositive patients, respectively. These data indicate the need to use at least two antigens in ELISA for the diagnosis of COVID-19 to improve assay sensitivity, especially at an early stage of the disease.

The available data demonstrating the influence of age on the B-cell immune response (in particular, on the rate of seroconversion and the titer of immunoglobulins) in COVID-19 patients remain inconclusive. A number of studies in elderly patients have reported a higher titer of antibodies of all classes [8, 46, 47]; however, there are studies that have reported no relationship between age and the B-cell response [48, 49]. There is no evidence of an effect of gender on the level of SARS-CoV-2-specific antibodies [47, 48].

**Fig. 6:**
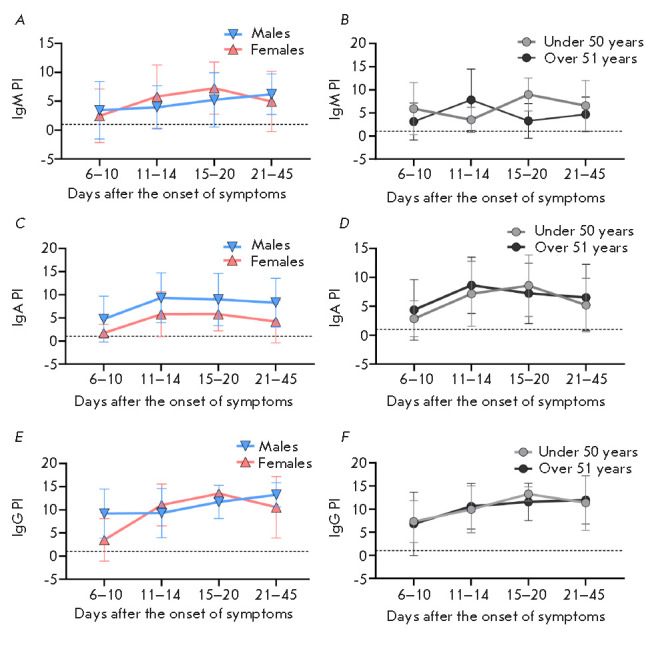
Distribution of the positivity index for the blood samples of patients, depending on days after the onset of symptoms in groups of males and females (*A, C, E*) and groups of patients of different ages (under 50 years and over 51 years) (*B, D, F*), calculated upon detection of SARS-CoV-2-specific class M (*A, B*), A (*C, D*), and G (*E, F*) immunoglobulins

We compared the blood levels of class M, A, and G immunoglobulins in patients of different age and gender groups from the cohort of hospitalized patients at different times after the onset of symptoms. For this purpose, we used ELISA and a mixture of recombinant proteins, SARS-CoV-2 S-protein RBD-SD1 and NTD fragments, and the recombinant N-protein as antigens. Comparison of PI values at each time interval using the nonparametric Mann–Whitney test did not reveal significant differences in the seroconversion rate between the study groups (*Fig. 6*).

To identify differences in the levels of class M, A, and G antibodies specific to different SARS-CoV-2 virus fragments in patients with differing severity of COVID-19, a group of outpatients (*n *= 50) who had had mild symptoms was additionally included in the cohort of patients who needed (or did not need) mechanical ventilation (mean time to hospitalization 21.2 days). Since the blood level of antibodies depends on the time from the onset of symptoms, for an accurate comparison, the hospitalized group included patients admitted 15–45 days (mean 21.8 days) after the onset of symptoms.

**Fig. 7:**
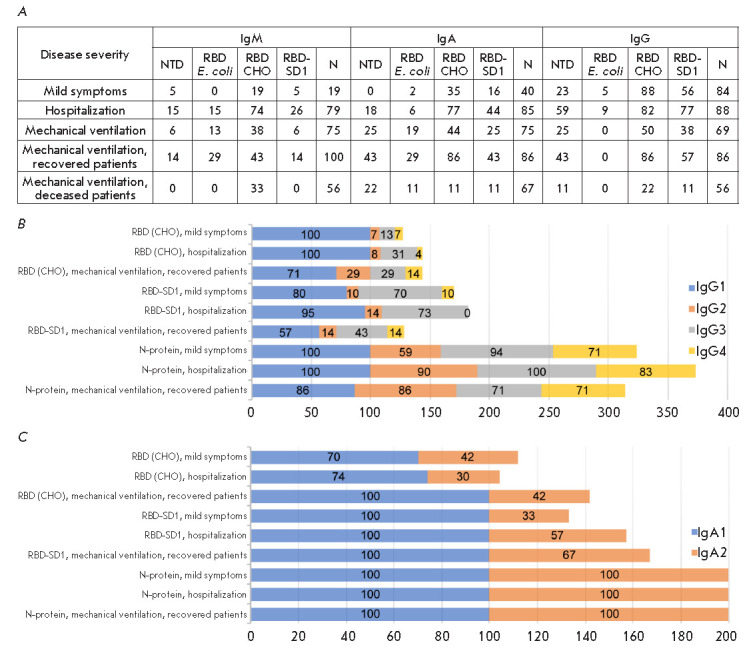
Changes in the occurrence rate of patients seropositive for immunoglobulins of various classes and subclasses specific to SARS-CoV-2 antigens in groups of patients with differing severity of COVID-19. (*A*) – occurrence rate (%) of patients seropositive for class M, A, and G immunoglobulins specific to NTD, RBD, RBD-SD1 antigens and the N-protein in the groups of patients with differing severity of COVID-19. (*B*) – occurrence rate (%) of patients seropositive for subclass G and A (*C*) immunoglobulins specific to RBD (CHO) and RBD-SD1 antigens and the N-protein in groups of patients with differing severity of COVID-19

An analysis of the occurrence rate of patients seropositive for class M or A immunoglobulins specific to one or more of the used antigens revealed a significant decrease in the rate in the group with mild symptoms compared to that of hospitalized patients seropositive for each antigen (*Fig. 7A*). In this group, there was also a decrease in the occurrence rate of class G immunoglobulins specific to linear RBD, NTD, and RBD-SD1 epitopes, while the occurrence rate of patients seropositive for RBD (CHO)- and N-protein-specific class G immunoglobulins did not change. In the group of patients on mechanical ventilation, the rate of patients seropositive for one or more classes of the antibodies under consideration was also reduced. However, this decrease was associated with a significant reduction in the number of seropositive patients in the subgroup of fatal cases, while these characteristics were similar in the subgroup of recovered patients and in the group of hospitalized patients.

An analysis of the levels of specific IgM, IgA, and IgG antibodies in serum-positive blood sera using the nonparametric Mann–Whitney test did not reveal a statistically significant effect of disease severity on the levels of SARS-CoV-2-specific antibodies. These results contradict some data indicating that the blood levels of immunoglobulins of various classes in severe patients are increased, while the content of antibodies is reduced in the group of asymptomatic or mild-symptoms patients [5, 8, 48, 50].

An analysis of the occurrence rate ratio of IgA and IgG subclasses in sera of appropriate seropositive samples (*Fig. 7B,C*) reveals a uniform induction of N-protein- specific immunoglobulin G subclasses G1–G4 and immunoglobulin A subclasses A1–A2 in groups of patients with differing severity of COVID-19, while G1, G3, and A1 are the main subclasses in the immune response to the S antigen. At more severe symptoms, the occurrence rate of S antigen-specific IgG1 antibodies is decreased, while that of IgA2, on the contrary, is increased. However, a correlation between the levels of the studied SARS-CoV-2-specific antibodies and disease severity has not been reliably established.

## CONCLUSION

By using the developed ELISA diagnostic kit based on recombinant antigens – SARS-CoV-2 virus protein fragments, we have reliably established the advantage of class A immunoglobulins as an early immunological criterion of the development of the disease. The spectrum of specificity of SARS-CoV-2-induced immunoglobulins in each patient depends on the time after infection and varies in the series of M, A, and G immunoglobulins from narrow to wide. We have also shown uneven induction of immunoglobulin subclasses, which depends on the antigen nature. The N-protein induces immunoglobulins G1–G4 and A1–A2 in equal proportions, while G1, G3, and A1 are the main subclasses in the immune response to the S-antigen. The ratio between N-specific subclasses remains almost unchanged in groups of patients with differing severity of COVID-19, but with a more severe course of the disease, the occurrence rate of S-specific IgG1 antibodies decreases, while that of IgA2, on the contrary, increases. However, no reliable correlation between the levels of the studied SARS-CoV-2-specific antibodies and disease severity has been revealed.

## Abbreviations

COVID-19: coronavirus disease 2019
SARS-CoV-2: severe acute respiratory syndrome coronavirus 2
PCR test: polymerase chain reaction test
ELISA: enzyme-linked immunosorbent assay
RBD-SD1: receptor-binding domain-subdomain 1
NTD: N-terminal domain
RBD: receptor binding domain
CHO cells: Chinese hamster ovary cells
